# (μ-Ethane-1,2-diamine-κ^2^ *N*:*N*′)bis­[dicarbon­yl(η^5^-cyclo­penta­dien­yl)iron(II)] bis­(tetra­fluorido­borate)

**DOI:** 10.1107/S1600536811010154

**Published:** 2011-03-26

**Authors:** Cyprian M. M’thiruaine, Holger B. Friedrich, Evans O. Changamu, Bernard Omondi

**Affiliations:** a School of Chemistry, University of KwaZulu-Natal, Westville Campus, Private Bag X54001, Durban 4000, South Africa; b Chemistry Department, Kenyatta University, PO Box 43844, Nairobi, Kenya; c Research Centre for Synthesis and Catalysis, Department of Chemistry, University of Johannesburg, PO Box 524 Auckland Park, Johannesburg 2006, South Africa

## Abstract

The asymmetric unit of the title compound, [Fe_2_(C_5_H_5_)_2_(C_2_H_8_N_2_)(CO)_4_](BF_4_)_2_, contains two half-cations, each located on a center of symmetry, and two tetra­fluorido­borate anions. The iron atoms adopt a three-legged piano-stool geometry. All amine H atoms are involved in N—H⋯F hydrogen bonds, which consolidate the crystal packing along with weak C—H⋯O and C—H⋯F inter­actions.

## Related literature

For the synthesis of the title compound and our previous work in this area, see: M’thiruaine *et al.* (2011 ▶). For related binuclear structures, see: Changamu & Friedrich (2008 ▶); Friedrich *et al.* (2005 ▶); Changamu, Friedrich, Howie & Rademeyer (2007 ▶); Changamu, Friedrich & Rademeyer (2007 ▶); Changamu *et al.* (2009 ▶).
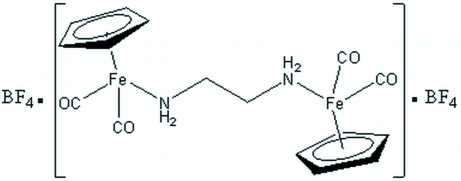


## Experimental

### 

#### Crystal data

[Fe_2_(C_5_H_5_)_2_(C_2_H_8_N_2_)(CO)_4_](BF_4_)_2_
*M* *_r_* = 587.64
Monoclinic, 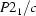

*a* = 11.5593 (7) Å
*b* = 15.5194 (9) Å
*c* = 12.4056 (8) Å
β = 95.774 (1)°
*V* = 2214.2 (2) Å^3^
*Z* = 4
Mo *K*α radiation
μ = 1.40 mm^−1^
*T* = 100 K
0.22 × 0.10 × 0.03 mm

#### Data collection

Bruker X8 APEXII 4K Kappa CCD diffractometer
Absorption correction: multi-scan (*SADABS*; Bruker, 2007 ▶) *T*_min_ = 0.748, *T*_max_ = 0.959
50791 measured reflections
5520 independent reflections
4548 reflections with *I* > 2σ(*I*)
*R*_int_ = 0.050

#### Refinement

*R*[*F* ^2^ > 2σ(*F* ^2^)] = 0.033
*wR*(*F* ^2^) = 0.084
*S* = 1.14
5520 reflections
307 parameters
5 restraints
H-atom parameters constrained
Δρ_max_ = 0.52 e Å^−3^
Δρ_min_ = −0.36 e Å^−3^

### 

Data collection: *APEX2* (Bruker, 2007 ▶); cell refinement: *SAINT-Plus* (Bruker, 2007 ▶); data reduction: *SAINT-Plus* and *XPREP* (Bruker, 2007 ▶); program(s) used to solve structure: *SHELXS97* (Sheldrick, 2008 ▶); program(s) used to refine structure: *SHELXL97* (Sheldrick, 2008 ▶); molecular graphics: *ORTEP-3* (Farrugia, 1997 ▶); software used to prepare material for publication: *WinGX* (Farrugia, 1999 ▶).

## Supplementary Material

Crystal structure: contains datablocks global, I. DOI: 10.1107/S1600536811010154/rz2564sup1.cif

Structure factors: contains datablocks I. DOI: 10.1107/S1600536811010154/rz2564Isup2.hkl

Additional supplementary materials:  crystallographic information; 3D view; checkCIF report

## Figures and Tables

**Table 1:** Hydrogen-bond geometry (Å, °)

*D*—H⋯*A*	*D*—H	H⋯*A*	*D*⋯*A*	*D*—H⋯*A*
N1—H1*A*⋯F8^i^	0.92	2.11	2.994 (2)	160
N1—H1*B*⋯F4^ii^	0.92	2.06	2.890 (2)	149
N2—H2*B*⋯F7^iii^	0.92	1.99	2.886 (2)	164
C3—H3⋯F1^iv^	1	2.36	3.326 (3)	163
C5—H5⋯F5^v^	1	2.39	3.216 (3)	139
C10—H10⋯O2^vi^	1	2.56	3.397 (3)	141
C10—H10⋯O3^vi^	1	2.57	3.326 (3)	132
C12—H12⋯F2^ii^	1	2.37	3.200 (3)	140

Symmetry codes: (i) 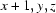; (ii) 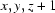; (iii) 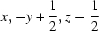; (iv) 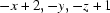; (v) 
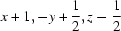
; (vi) 
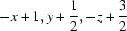
.

**Fig. 1.**
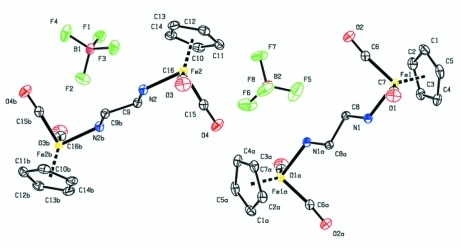
The asymmetric unit of the title compound with displacement ellipsoids drawn at the 50% probability level. Hydrogen atoms are omitted for clarity. Symmetry codes: (a) 2 - x, -y, 2 - z; (b) 1 - x, 1 - y, 1 - z

**Fig. 2.**
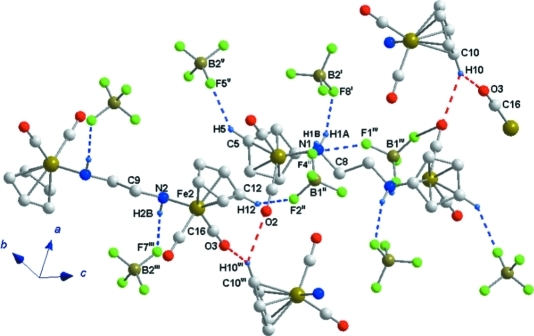
Partial crystal packing of the title compound showing, N–H···F, C–H···O and C–H···F intermolecular hydrogen interactions (dashed lines). Symmetry codes: (i) x + 1, y, z; (ii) x, y, z + 1; (iii) x, -y + 1/2, z - 1/2; (iv) -x + 2, -y, -z + 1; (v) x + 1, -y + 1/2, z - 1/2; (vi) -x + 1, y + 1/2, -z + 3/2.

## supplementary crystallographic information

### Comment

The title compound is a water-soluble organometallic compound which was prepared as part of our ongoing study on functionalized alkyl transition metal complexes (M'thiruaine *et al.*, 2011; Changamu & Friedrich 2008; Friedrich *et al.*, 2005; Changamu, Friedrich, Howie, & Rademeyer, 2007; Changamu, Friedrich & Rademeyer, 2007; Changamu, Friedrich & Rademeyer, 2007; Changamu *et al.*, 2009). The asymmetric unit (Fig. 1) has two half-cations, each located on a center of symmetry, and two tetrafluoroborate counteranions. The Fe metals are coordinated in three-legged piano-stool fashion with the cyclopentadienyl rings occupying the apical positions and two carbonyl ligands and one ethylenediamine nitrogen occupying the basal positions. The cyclopentadienyl ligands are *trans* to each other and the ethylenediamine ligands display a *trans* conformation. The Fe–*Cg* distances are 1.7123 (3) and 1.7103 (3) Å for atoms Fe1 and Fe2, respectively (*Cg* is the centroid of the cyclopentadienyl rings). In the crystal structure, the amine hydrogen atoms and the fluorine atoms of the tetrafluoroborate anions are engaged in three N—H···F intermolecular interactions (Table 1). Three H atoms of the cyclopentadienyl rings are also involved in C—H···F intermolecular interactions. Three C—H···O hydrogen bonds then complete the stabilization of the crystal lattice (Fig. 2).

### Experimental

The compound was synthesized by reacting ethylenediamine with two equivalents of [CpFe(CO)_2_(Et_2_O)]BF_4_, according to the literature procedure (M'thiruaine *et al.*, 2011). Yield: 0.339 g, 78%; m.p: dec >120°C. Spectroscopic analysis: ^1^H NMR (400 MHz, acetone-d6): δ 5.48 (s, 10H, Cp), 3.33 (s, 4H, NH_2_), 2.58 (s, 4H, CH_2_). ^13^C NMR (400 MHz, acetone-d6): δ 87.43 (Cp), 54.50 (CH_2_), 211.92 (CO). Ms: m/*z* 500.8 [[{CpFe(CO)_2_}_2_NH_2_CH_2_CH_2_NH_2_]BF_4_]^+^; 236.7 [CpFe(CO)_2_NH_2_CH_2_CH_2_NH_2_]^+^; 220.9 [CpFe(CO)_2_NH_2_CH_2_CH_2_]^+^; 206.6 [CpFe(CO)_2_NH_2_CH_2_]^+^; 194.6 [CpFe(CO)_2_NH_2_^+^_2_H]^+^; 176.6 [CpFe(CO)_2_]^+^. Elemental analysis: calculated for C_16_H_18_B_2_F_8_Fe_2_N_2_O_4_: C, 32.65; H, 3.06; N, 4.76. Found: C, 32.96; H, 3.28; N, 5.02%. IR (solid state): ν(CO) 2054, 2001 cm^-1^; ν(NH) 3315, 3276 cm^-1^.

### Refinement

All H atoms were placed in geometrically idealized positions and constrained to ride on their parent atoms, with C—H = 0.99–1.00 Å, N—H = 0.92 Å, and with *U*_iso_(H) = 1.2*U*_eq_(C, N). In the final refinement cycles restraints were applied to the anisotropic displacement parameters of atoms Fe1 and C7 (SIMU and DELU instructions in *SHELXL97*, Sheldrick, 2008). Two reflections (-8 2 2, 5 2 6) were identified as outliers and removed from the refinement.

### Crystal data

**Table:**

[Fe_2_(C_5_H_5_)_2_(C_2_H_8_N_2_)(CO)_4_](BF_4_)_2_	*F*(000) = 1176
*M**_r_* = 587.64	*D*_x_ = 1.763 Mg m^−^^3^
Monoclinic, *P*2_1_/*c*	Mo *K*α radiation, λ = 0.71073 Å
Hall symbol: -P 2ybc	Cell parameters from 51845 reflections
*a* = 11.5593 (7) Å	θ = 1.8–28.3°
*b* = 15.5194 (9) Å	µ = 1.40 mm^−^^1^
*c* = 12.4056 (8) Å	*T* = 100 K
β = 95.774 (1)°	Plate, yellow
*V* = 2214.2 (2) Å^3^	0.22 × 0.1 × 0.03 mm
*Z* = 4	

### Data collection

**Table:**

Bruker X8 APEXII 4K Kappa CCD diffractometer	4548 reflections with *I* > 2σ(*I*)
graphite	*R*_int_ = 0.050
φ and ω scans	θ_max_ = 28.3°, θ_min_ = 1.8°
Absorption correction: multi-scan (*SADABS*; Bruker, 2007)	*h* = −15→15
*T*_min_ = 0.748, *T*_max_ = 0.959	*k* = −20→20
50791 measured reflections	*l* = −16→16
5520 independent reflections	

### Refinement

**Table:**

Refinement on *F*^2^	Primary atom site location: structure-invariant direct methods
Least-squares matrix: full	Secondary atom site location: difference Fourier map
*R*[*F*^2^ > 2σ(*F*^2^)] = 0.033	Hydrogen site location: inferred from neighbouring sites
*wR*(*F*^2^) = 0.084	H-atom parameters constrained
*S* = 1.14	*w* = 1/[σ^2^(*F*_o_^2^) + (0.0322*P*)^2^ + 1.9*P*] where *P* = (*F*_o_^2^ + 2*F*_c_^2^)/3
5520 reflections	(Δ/σ)_max_ = 0.012
307 parameters	Δρ_max_ = 0.52 e Å^−^^3^
5 restraints	Δρ_min_ = −0.36 e Å^−^^3^

### Special details

**Table:**

Experimental. The intensity data was collected on a Bruker X8 Apex 4 K CCD diffractometer using an exposure time of 20 sec/per frame. A total of 2220 frames were collected with a frame width of 0.5° covering upto θ = 28.31° with 99.9% completeness accomplished.Two reflections omited. -8 2 2 and 5 2 6 Reason - affecting number of intensities with I.LT - 2*sig(I)
Geometry. All e.s.d.'s (except the e.s.d. in the dihedral angle between two l.s. planes) are estimated using the full covariance matrix. The cell e.s.d.'s are taken into account individually in the estimation of e.s.d.'s in distances, angles and torsion angles; correlations between e.s.d.'s in cell parameters are only used when they are defined by crystal symmetry. An approximate (isotropic) treatment of cell e.s.d.'s is used for estimating e.s.d.'s involving l.s. planes.
Refinement. Refinement of *F*^2^ against ALL reflections. The weighted *R*-factor *wR* and goodness of fit *S* are based on *F*^2^, conventional *R*-factors *R* are based on *F*, with *F* set to zero for negative *F*^2^. The threshold expression of *F*^2^ > σ(*F*^2^) is used only for calculating *R*-factors(gt) *etc*. and is not relevant to the choice of reflections for refinement. *R*-factors based on *F*^2^ are statistically about twice as large as those based on *F*, and *R*- factors based on ALL data will be even larger.

### Fractional atomic coordinates and isotropic or equivalent isotropic displacement parameters (Å^2^)

**Table:**

	*x*	*y*	*z*	*U*_iso_*/*U*_eq_	
C1	0.9375 (2)	0.08248 (16)	0.57592 (18)	0.0247 (5)	
H1	0.8639	0.0936	0.5285	0.03*	
C2	0.9648 (2)	0.00609 (14)	0.63716 (18)	0.0219 (5)	
H2	0.9135	−0.0454	0.641	0.026*	
C3	1.07764 (19)	0.01558 (14)	0.68957 (18)	0.0203 (4)	
H3	1.1197	−0.0279	0.7384	0.024*	
C4	1.1215 (2)	0.09711 (14)	0.66286 (19)	0.0219 (5)	
H4	1.2001	0.1208	0.6878	0.026*	
C5	1.0341 (2)	0.13820 (15)	0.59066 (19)	0.0248 (5)	
H5	1.0408	0.1959	0.5559	0.03*	
C6	0.81634 (18)	0.10642 (13)	0.75085 (17)	0.0162 (4)	
C7	0.97598 (19)	0.22773 (14)	0.76730 (18)	0.0194 (4)	
C8	0.97213 (17)	0.00688 (13)	0.94213 (16)	0.0144 (4)	
H8A	0.9856	−0.0445	0.8978	0.017*	
H8B	0.8871	0.0144	0.9427	0.017*	
N1	1.02188 (15)	0.08372 (11)	0.89374 (14)	0.0156 (3)	
H1A	1.1013	0.0767	0.8992	0.019*	
H1B	1.0071	0.1304	0.936	0.019*	
O1	0.97866 (16)	0.29991 (10)	0.78238 (15)	0.0309 (4)	
O2	0.71853 (14)	0.09930 (11)	0.74774 (14)	0.0250 (4)	
Fe1	0.96912 (2)	0.114343 (18)	0.73886 (2)	0.01336 (8)	
C9	0.48038 (18)	0.49238 (13)	0.55612 (17)	0.0166 (4)	
H9A	0.5125	0.5381	0.6062	0.02*	
H9B	0.3945	0.4954	0.552	0.02*	
C10	0.5143 (2)	0.48047 (14)	0.85356 (18)	0.0223 (5)	
H10	0.469	0.5355	0.8478	0.027*	
C11	0.4862 (2)	0.40502 (15)	0.91205 (18)	0.0213 (4)	
H11	0.4181	0.3982	0.9551	0.026*	
C12	0.5737 (2)	0.34334 (15)	0.90177 (18)	0.0226 (5)	
H12	0.5778	0.2848	0.9355	0.027*	
C13	0.6554 (2)	0.37873 (16)	0.83542 (19)	0.0250 (5)	
H13	0.7271	0.3497	0.8145	0.03*	
C14	0.6186 (2)	0.46380 (15)	0.80752 (19)	0.0238 (5)	
H14	0.6593	0.5048	0.7619	0.029*	
C15	0.33575 (18)	0.38898 (13)	0.72337 (17)	0.0172 (4)	
C16	0.4813 (2)	0.26115 (14)	0.71961 (19)	0.0217 (4)	
N2	0.52009 (16)	0.40719 (11)	0.59844 (14)	0.0169 (4)	
H2A	0.5991	0.4041	0.5946	0.02*	
H2B	0.4862	0.366	0.5522	0.02*	
O3	0.47605 (17)	0.18899 (11)	0.70654 (16)	0.0352 (4)	
O4	0.23808 (14)	0.40008 (11)	0.71176 (15)	0.0278 (4)	
Fe2	0.48971 (2)	0.374292 (18)	0.74962 (2)	0.01372 (8)	
B1	0.7830 (2)	0.17693 (15)	0.0504 (2)	0.0185 (5)	
F1	0.78888 (12)	0.09908 (9)	0.10823 (12)	0.0298 (3)	
F2	0.70586 (14)	0.16543 (10)	−0.04143 (12)	0.0349 (4)	
F3	0.74205 (13)	0.24107 (9)	0.11366 (13)	0.0328 (3)	
F4	0.89235 (13)	0.19761 (10)	0.02152 (14)	0.0368 (4)	
B2	0.2759 (2)	0.19786 (17)	0.9296 (2)	0.0226 (5)	
F5	0.18759 (16)	0.21368 (12)	0.99271 (18)	0.0577 (6)	
F6	0.25526 (17)	0.23972 (12)	0.83285 (16)	0.0568 (6)	
F7	0.38195 (13)	0.22456 (10)	0.98276 (14)	0.0387 (4)	
F8	0.27978 (13)	0.10907 (10)	0.90999 (15)	0.0384 (4)	

### Atomic displacement parameters (Å^2^)

**Table:**

	*U*^11^	*U*^22^	*U*^33^	*U*^12^	*U*^13^	*U*^23^
C1	0.0269 (12)	0.0314 (13)	0.0155 (10)	0.0102 (10)	0.0010 (9)	−0.0019 (9)
C2	0.0274 (11)	0.0177 (10)	0.0219 (11)	0.0023 (9)	0.0082 (9)	−0.0068 (8)
C3	0.0235 (11)	0.0181 (10)	0.0204 (10)	0.0085 (9)	0.0080 (9)	0.0014 (8)
C4	0.0210 (11)	0.0205 (11)	0.0259 (11)	0.0027 (9)	0.0106 (9)	0.0011 (9)
C5	0.0352 (13)	0.0197 (11)	0.0218 (11)	0.0091 (9)	0.0147 (10)	0.0067 (8)
C6	0.0188 (8)	0.0125 (9)	0.0179 (10)	0.0011 (8)	0.0038 (8)	−0.0006 (7)
C7	0.0204 (10)	0.0158 (8)	0.0217 (10)	0.0009 (8)	0.0008 (9)	0.0010 (8)
C8	0.0139 (9)	0.0130 (9)	0.0161 (10)	−0.0016 (7)	0.0008 (8)	0.0004 (7)
N1	0.0165 (8)	0.0145 (8)	0.0162 (8)	−0.0017 (7)	0.0030 (7)	0.0012 (7)
O1	0.0383 (10)	0.0147 (8)	0.0383 (10)	0.0016 (7)	−0.0024 (8)	−0.0015 (7)
O2	0.0166 (8)	0.0261 (9)	0.0322 (9)	−0.0001 (6)	0.0023 (7)	0.0004 (7)
Fe1	0.01438 (14)	0.01098 (13)	0.01499 (14)	0.00171 (11)	0.00288 (11)	0.00099 (11)
C9	0.0164 (9)	0.0152 (10)	0.0185 (10)	0.0011 (8)	0.0035 (8)	0.0027 (8)
C10	0.0266 (11)	0.0154 (10)	0.0233 (11)	−0.0021 (9)	−0.0054 (9)	−0.0029 (8)
C11	0.0245 (11)	0.0220 (11)	0.0173 (10)	−0.0038 (9)	0.0021 (9)	−0.0011 (8)
C12	0.0270 (12)	0.0194 (11)	0.0203 (11)	0.0003 (9)	−0.0035 (9)	0.0046 (8)
C13	0.0166 (10)	0.0308 (12)	0.0265 (12)	0.0002 (9)	−0.0036 (9)	0.0033 (10)
C14	0.0216 (11)	0.0241 (11)	0.0243 (11)	−0.0112 (8)	−0.0044 (9)	0.0053 (9)
C15	0.0175 (7)	0.0140 (9)	0.0204 (10)	0.0007 (8)	0.0034 (8)	0.0031 (8)
C16	0.0224 (11)	0.0148 (8)	0.0279 (12)	0.0023 (8)	0.0017 (9)	0.0006 (8)
N2	0.0187 (9)	0.0143 (8)	0.0181 (8)	0.0024 (7)	0.0039 (7)	0.0028 (7)
O3	0.0412 (11)	0.0154 (8)	0.0480 (12)	0.0028 (8)	−0.0006 (9)	−0.0020 (8)
O4	0.0175 (8)	0.0254 (9)	0.0410 (10)	0.0022 (7)	0.0048 (7)	0.0044 (7)
Fe2	0.01287 (14)	0.01104 (14)	0.01749 (15)	−0.00015 (11)	0.00264 (11)	0.00186 (11)
B1	0.0181 (11)	0.0146 (11)	0.0237 (12)	−0.0003 (9)	0.0064 (9)	−0.0038 (9)
F1	0.0247 (7)	0.0270 (7)	0.0380 (8)	0.0034 (6)	0.0049 (6)	0.0090 (6)
F2	0.0416 (9)	0.0320 (8)	0.0291 (8)	−0.0016 (7)	−0.0064 (7)	−0.0019 (6)
F3	0.0315 (8)	0.0280 (8)	0.0411 (9)	0.0001 (6)	0.0145 (7)	−0.0151 (6)
F4	0.0300 (8)	0.0287 (8)	0.0559 (10)	−0.0103 (6)	0.0247 (7)	−0.0126 (7)
B2	0.0151 (11)	0.0204 (12)	0.0316 (14)	−0.0058 (9)	−0.0009 (10)	0.0064 (10)
F5	0.0409 (10)	0.0524 (12)	0.0845 (15)	−0.0158 (9)	0.0292 (10)	−0.0334 (10)
F6	0.0543 (11)	0.0554 (12)	0.0550 (12)	−0.0269 (9)	−0.0226 (9)	0.0326 (9)
F7	0.0267 (8)	0.0353 (9)	0.0502 (10)	−0.0140 (7)	−0.0152 (7)	0.0142 (7)
F8	0.0213 (7)	0.0241 (8)	0.0684 (12)	0.0012 (6)	−0.0016 (7)	0.0004 (7)

### Geometric parameters (Å, °)

**Table:**

C1—C5	1.409 (4)	C10—C14	1.409 (3)
C1—C2	1.426 (3)	C10—C11	1.432 (3)
C1—Fe1	2.077 (2)	C10—Fe2	2.094 (2)
C1—H1	1	C10—H10	1
C2—C3	1.405 (3)	C11—C12	1.407 (3)
C2—Fe1	2.099 (2)	C11—Fe2	2.075 (2)
C2—H2	1	C11—H11	1
C3—C4	1.415 (3)	C12—C13	1.424 (3)
C3—Fe1	2.110 (2)	C12—Fe2	2.090 (2)
C3—H3	1	C12—H12	1
C4—C5	1.431 (3)	C13—C14	1.419 (3)
C4—Fe1	2.097 (2)	C13—Fe2	2.097 (2)
C4—H4	1	C13—H13	1
C5—Fe1	2.088 (2)	C14—Fe2	2.109 (2)
C5—H5	1	C14—H14	1
C6—O2	1.133 (3)	C15—O4	1.137 (3)
C6—Fe1	1.791 (2)	C15—Fe2	1.791 (2)
C7—O1	1.136 (3)	C16—O3	1.132 (3)
C7—Fe1	1.795 (2)	C16—Fe2	1.796 (2)
C8—N1	1.477 (2)	N2—Fe2	2.0085 (18)
C8—C8^i^	1.528 (4)	N2—H2A	0.92
C8—H8A	0.99	N2—H2B	0.92
C8—H8B	0.99	B1—F3	1.381 (3)
N1—Fe1	2.0134 (17)	B1—F4	1.385 (3)
N1—H1A	0.92	B1—F2	1.386 (3)
N1—H1B	0.92	B1—F1	1.404 (3)
C9—N2	1.478 (3)	B2—F6	1.364 (3)
C9—C9^ii^	1.526 (4)	B2—F5	1.370 (3)
C9—H9A	0.99	B2—F7	1.395 (3)
C9—H9B	0.99	B2—F8	1.401 (3)
			
C5—C1—C2	108.1 (2)	C14—C10—C11	107.6 (2)
C5—C1—Fe1	70.63 (13)	C14—C10—Fe2	71.01 (13)
C2—C1—Fe1	70.84 (13)	C11—C10—Fe2	69.21 (12)
C5—C1—H1	125.9	C14—C10—H10	126.2
C2—C1—H1	125.9	C11—C10—H10	126.2
Fe1—C1—H1	125.9	Fe2—C10—H10	126.2
C3—C2—C1	107.7 (2)	C12—C11—C10	108.0 (2)
C3—C2—Fe1	70.92 (12)	C12—C11—Fe2	70.81 (13)
C1—C2—Fe1	69.23 (13)	C10—C11—Fe2	70.62 (12)
C3—C2—H2	126.1	C12—C11—H11	126
C1—C2—H2	126.1	C10—C11—H11	126
Fe1—C2—H2	126.1	Fe2—C11—H11	126
C2—C3—C4	108.8 (2)	C11—C12—C13	108.3 (2)
C2—C3—Fe1	70.07 (12)	C11—C12—Fe2	69.69 (13)
C4—C3—Fe1	69.85 (12)	C13—C12—Fe2	70.38 (13)
C2—C3—H3	125.6	C11—C12—H12	125.8
C4—C3—H3	125.6	C13—C12—H12	125.8
Fe1—C3—H3	125.6	Fe2—C12—H12	125.8
C3—C4—C5	107.4 (2)	C14—C13—C12	107.4 (2)
C3—C4—Fe1	70.85 (12)	C14—C13—Fe2	70.76 (13)
C5—C4—Fe1	69.68 (12)	C12—C13—Fe2	69.84 (13)
C3—C4—H4	126.3	C14—C13—H13	126.3
C5—C4—H4	126.3	C12—C13—H13	126.3
Fe1—C4—H4	126.3	Fe2—C13—H13	126.3
C1—C5—C4	107.9 (2)	C10—C14—C13	108.6 (2)
C1—C5—Fe1	69.82 (13)	C10—C14—Fe2	69.82 (13)
C4—C5—Fe1	70.33 (12)	C13—C14—Fe2	69.80 (13)
C1—C5—H5	126	C10—C14—H14	125.7
C4—C5—H5	126	C13—C14—H14	125.7
Fe1—C5—H5	126	Fe2—C14—H14	125.7
O2—C6—Fe1	173.12 (19)	O4—C15—Fe2	176.5 (2)
O1—C7—Fe1	178.0 (2)	O3—C16—Fe2	176.3 (2)
N1—C8—C8^i^	110.5 (2)	C9—N2—Fe2	118.79 (13)
N1—C8—H8A	109.6	C9—N2—H2A	107.6
C8^i^—C8—H8A	109.6	Fe2—N2—H2A	107.6
N1—C8—H8B	109.6	C9—N2—H2B	107.6
C8^i^—C8—H8B	109.6	Fe2—N2—H2B	107.6
H8A—C8—H8B	108.1	H2A—N2—H2B	107
C8—N1—Fe1	119.03 (13)	C15—Fe2—C16	93.13 (10)
C8—N1—H1A	107.6	C15—Fe2—N2	93.54 (8)
Fe1—N1—H1A	107.6	C16—Fe2—N2	93.78 (9)
C8—N1—H1B	107.6	C15—Fe2—C11	91.78 (9)
Fe1—N1—H1B	107.6	C16—Fe2—C11	114.84 (10)
H1A—N1—H1B	107	N2—Fe2—C11	150.54 (8)
C6—Fe1—C7	94.32 (10)	C15—Fe2—C12	123.78 (9)
C6—Fe1—N1	96.38 (8)	C16—Fe2—C12	88.46 (10)
C7—Fe1—N1	92.33 (9)	N2—Fe2—C12	142.46 (9)
C6—Fe1—C1	89.22 (9)	C11—Fe2—C12	39.50 (9)
C7—Fe1—C1	115.13 (10)	C15—Fe2—C10	94.73 (9)
N1—Fe1—C1	151.53 (9)	C16—Fe2—C10	153.95 (10)
C6—Fe1—C5	122.03 (10)	N2—Fe2—C10	110.45 (8)
C7—Fe1—C5	89.23 (10)	C11—Fe2—C10	40.17 (9)
N1—Fe1—C5	141.34 (9)	C12—Fe2—C10	66.59 (9)
C1—Fe1—C5	39.55 (10)	C15—Fe2—C13	158.17 (10)
C6—Fe1—C4	155.27 (10)	C16—Fe2—C13	99.50 (10)
C7—Fe1—C4	101.01 (9)	N2—Fe2—C13	103.24 (9)
N1—Fe1—C4	102.21 (8)	C11—Fe2—C13	66.78 (9)
C1—Fe1—C4	66.76 (9)	C12—Fe2—C13	39.78 (9)
C5—Fe1—C4	39.98 (9)	C10—Fe2—C13	66.50 (10)
C6—Fe1—C2	91.78 (9)	C15—Fe2—C14	129.26 (10)
C7—Fe1—C2	154.28 (10)	C16—Fe2—C14	137.42 (10)
N1—Fe1—C2	111.80 (8)	N2—Fe2—C14	88.09 (8)
C1—Fe1—C2	39.93 (9)	C11—Fe2—C14	66.43 (9)
C5—Fe1—C2	66.51 (9)	C12—Fe2—C14	66.17 (9)
C4—Fe1—C2	66.28 (9)	C10—Fe2—C14	39.17 (9)
C6—Fe1—C3	126.45 (9)	C13—Fe2—C14	39.44 (9)
C7—Fe1—C3	138.89 (10)	F3—B1—F4	110.73 (19)
N1—Fe1—C3	88.46 (8)	F3—B1—F2	109.3 (2)
C1—Fe1—C3	66.20 (9)	F4—B1—F2	110.2 (2)
C5—Fe1—C3	66.20 (8)	F3—B1—F1	109.35 (19)
C4—Fe1—C3	39.30 (8)	F4—B1—F1	109.47 (19)
C2—Fe1—C3	39.01 (9)	F2—B1—F1	107.66 (18)
N2—C9—C9^ii^	110.6 (2)	F6—B2—F5	110.0 (2)
N2—C9—H9A	109.5	F6—B2—F7	110.02 (19)
C9^ii^—C9—H9A	109.5	F5—B2—F7	110.3 (2)
N2—C9—H9B	109.5	F6—B2—F8	108.8 (2)
C9^ii^—C9—H9B	109.5	F5—B2—F8	108.30 (19)
H9A—C9—H9B	108.1	F7—B2—F8	109.4 (2)
			
C5—C1—C2—C3	0.3 (2)	C14—C10—C11—C12	0.3 (3)
Fe1—C1—C2—C3	−60.77 (15)	Fe2—C10—C11—C12	61.28 (16)
C5—C1—C2—Fe1	61.11 (15)	C14—C10—C11—Fe2	−60.95 (16)
C1—C2—C3—C4	0.4 (2)	C10—C11—C12—C13	−1.2 (3)
Fe1—C2—C3—C4	−59.27 (15)	Fe2—C11—C12—C13	60.01 (16)
C1—C2—C3—Fe1	59.70 (15)	C10—C11—C12—Fe2	−61.17 (15)
C2—C3—C4—C5	−1.0 (2)	C11—C12—C13—C14	1.5 (3)
Fe1—C3—C4—C5	−60.42 (15)	Fe2—C12—C13—C14	61.11 (16)
C2—C3—C4—Fe1	59.41 (15)	C11—C12—C13—Fe2	−59.58 (16)
C2—C1—C5—C4	−1.0 (2)	C11—C10—C14—C13	0.6 (3)
Fe1—C1—C5—C4	60.28 (16)	Fe2—C10—C14—C13	−59.18 (16)
C2—C1—C5—Fe1	−61.24 (15)	C11—C10—C14—Fe2	59.80 (15)
C3—C4—C5—C1	1.2 (2)	C12—C13—C14—C10	−1.3 (3)
Fe1—C4—C5—C1	−59.95 (15)	Fe2—C13—C14—C10	59.20 (16)
C3—C4—C5—Fe1	61.17 (15)	C12—C13—C14—Fe2	−60.52 (16)
C8^i^—C8—N1—Fe1	177.40 (16)	C9^ii^—C9—N2—Fe2	175.74 (18)
C8—N1—Fe1—C6	48.97 (16)	C9—N2—Fe2—C15	57.08 (16)
C8—N1—Fe1—C7	143.59 (16)	C9—N2—Fe2—C16	150.45 (17)
C8—N1—Fe1—C1	−51.2 (2)	C9—N2—Fe2—C11	−42.9 (3)
C8—N1—Fe1—C5	−124.73 (16)	C9—N2—Fe2—C12	−117.16 (17)
C8—N1—Fe1—C4	−114.62 (15)	C9—N2—Fe2—C10	−39.31 (18)
C8—N1—Fe1—C2	−45.53 (17)	C9—N2—Fe2—C13	−108.86 (16)
C8—N1—Fe1—C3	−77.54 (15)	C9—N2—Fe2—C14	−72.14 (16)
C5—C1—Fe1—C6	148.16 (14)	C12—C11—Fe2—C15	146.81 (14)
C2—C1—Fe1—C6	−93.72 (14)	C10—C11—Fe2—C15	−95.23 (14)
C5—C1—Fe1—C7	53.76 (16)	C12—C11—Fe2—C16	52.54 (16)
C2—C1—Fe1—C7	171.87 (14)	C10—C11—Fe2—C16	170.50 (14)
C5—C1—Fe1—N1	−109.84 (19)	C12—C11—Fe2—N2	−112.77 (19)
C2—C1—Fe1—N1	8.3 (3)	C10—C11—Fe2—N2	5.2 (2)
C2—C1—Fe1—C5	118.12 (19)	C10—C11—Fe2—C12	117.96 (19)
C5—C1—Fe1—C4	−37.85 (13)	C12—C11—Fe2—C10	−117.96 (19)
C2—C1—Fe1—C4	80.26 (14)	C12—C11—Fe2—C13	−37.42 (14)
C5—C1—Fe1—C2	−118.12 (19)	C10—C11—Fe2—C13	80.53 (15)
C5—C1—Fe1—C3	−80.88 (14)	C12—C11—Fe2—C14	−80.56 (15)
C2—C1—Fe1—C3	37.23 (13)	C10—C11—Fe2—C14	37.39 (13)
C1—C5—Fe1—C6	−38.47 (17)	C11—C12—Fe2—C15	−41.17 (17)
C4—C5—Fe1—C6	−157.13 (14)	C13—C12—Fe2—C15	−160.38 (14)
C1—C5—Fe1—C7	−133.09 (15)	C11—C12—Fe2—C16	−133.90 (15)
C4—C5—Fe1—C7	108.25 (15)	C13—C12—Fe2—C16	106.89 (15)
C1—C5—Fe1—N1	134.14 (15)	C11—C12—Fe2—N2	131.91 (15)
C4—C5—Fe1—N1	15.5 (2)	C13—C12—Fe2—N2	12.7 (2)
C4—C5—Fe1—C1	−118.66 (19)	C13—C12—Fe2—C11	−119.2 (2)
C1—C5—Fe1—C4	118.66 (19)	C11—C12—Fe2—C10	38.38 (13)
C1—C5—Fe1—C2	38.12 (13)	C13—C12—Fe2—C10	−80.83 (15)
C4—C5—Fe1—C2	−80.54 (15)	C11—C12—Fe2—C13	119.2 (2)
C1—C5—Fe1—C3	80.87 (14)	C11—C12—Fe2—C14	81.30 (15)
C4—C5—Fe1—C3	−37.79 (13)	C13—C12—Fe2—C14	−37.92 (14)
C3—C4—Fe1—C6	−65.8 (3)	C14—C10—Fe2—C15	−154.65 (14)
C5—C4—Fe1—C6	52.0 (3)	C11—C10—Fe2—C15	87.15 (14)
C3—C4—Fe1—C7	166.93 (14)	C14—C10—Fe2—C16	98.3 (2)
C5—C4—Fe1—C7	−75.33 (15)	C11—C10—Fe2—C16	−19.9 (3)
C3—C4—Fe1—N1	72.08 (14)	C14—C10—Fe2—N2	−59.08 (15)
C5—C4—Fe1—N1	−170.18 (13)	C11—C10—Fe2—N2	−177.28 (13)
C3—C4—Fe1—C1	−80.29 (15)	C14—C10—Fe2—C11	118.21 (19)
C5—C4—Fe1—C1	37.45 (14)	C14—C10—Fe2—C12	80.45 (14)
C3—C4—Fe1—C5	−117.7 (2)	C11—C10—Fe2—C12	−37.75 (14)
C3—C4—Fe1—C2	−36.57 (14)	C14—C10—Fe2—C13	36.92 (13)
C5—C4—Fe1—C2	81.17 (15)	C11—C10—Fe2—C13	−81.29 (15)
C5—C4—Fe1—C3	117.7 (2)	C11—C10—Fe2—C14	−118.21 (19)
C3—C2—Fe1—C6	−154.94 (14)	C14—C13—Fe2—C15	−69.2 (3)
C1—C2—Fe1—C6	86.64 (14)	C12—C13—Fe2—C15	48.6 (3)
C3—C2—Fe1—C7	101.3 (2)	C14—C13—Fe2—C16	166.33 (15)
C1—C2—Fe1—C7	−17.2 (3)	C12—C13—Fe2—C16	−75.89 (15)
C3—C2—Fe1—N1	−57.35 (14)	C14—C13—Fe2—N2	70.13 (15)
C1—C2—Fe1—N1	−175.76 (13)	C12—C13—Fe2—N2	−172.10 (13)
C3—C2—Fe1—C1	118.42 (19)	C14—C13—Fe2—C11	−80.61 (15)
C3—C2—Fe1—C5	80.65 (14)	C12—C13—Fe2—C11	37.17 (14)
C1—C2—Fe1—C5	−37.76 (14)	C14—C13—Fe2—C12	−117.8 (2)
C3—C2—Fe1—C4	36.84 (13)	C14—C13—Fe2—C10	−36.67 (14)
C1—C2—Fe1—C4	−81.57 (15)	C12—C13—Fe2—C10	81.10 (15)
C1—C2—Fe1—C3	−118.42 (19)	C12—C13—Fe2—C14	117.8 (2)
C2—C3—Fe1—C6	31.76 (17)	C10—C14—Fe2—C15	33.45 (18)
C4—C3—Fe1—C6	151.69 (14)	C13—C14—Fe2—C15	153.33 (15)
C2—C3—Fe1—C7	−139.66 (16)	C10—C14—Fe2—C16	−140.03 (16)
C4—C3—Fe1—C7	−19.7 (2)	C13—C14—Fe2—C16	−20.1 (2)
C2—C3—Fe1—N1	128.55 (13)	C10—C14—Fe2—N2	126.46 (14)
C4—C3—Fe1—N1	−111.52 (14)	C13—C14—Fe2—N2	−113.66 (14)
C2—C3—Fe1—C1	−38.10 (14)	C10—C14—Fe2—C11	−38.33 (13)
C4—C3—Fe1—C1	81.83 (15)	C13—C14—Fe2—C11	81.55 (15)
C2—C3—Fe1—C5	−81.50 (15)	C10—C14—Fe2—C12	−81.64 (15)
C4—C3—Fe1—C5	38.43 (15)	C13—C14—Fe2—C12	38.24 (14)
C2—C3—Fe1—C4	−119.93 (19)	C13—C14—Fe2—C10	119.9 (2)
C4—C3—Fe1—C2	119.93 (19)	C10—C14—Fe2—C13	−119.9 (2)

Symmetry codes: (i) −*x*+2, −*y*, −*z*+2; (ii) −*x*+1, −*y*+1, −*z*+1.

### Hydrogen-bond geometry (Å, °)

**Table:**

*D*—H···*A*	*D*—H	H···*A*	*D*···*A*	*D*—H···*A*
N1—H1A···F8^iii^	0.92	2.11	2.994 (2)	160
N1—H1B···F4^iv^	0.92	2.06	2.890 (2)	149
N2—H2B···F7^v^	0.92	1.99	2.886 (2)	164
C3—H3···F1^vi^	1	2.36	3.326 (3)	163
C5—H5···F5^vii^	1	2.39	3.216 (3)	139
C10—H10···O2^viii^	1	2.56	3.397 (3)	141
C10—H10···O3^viii^	1	2.57	3.326 (3)	132
C12—H12···F2^iv^	1	2.37	3.200 (3)	140

Symmetry codes: (iii) *x*+1, *y*, *z*; (iv) *x*, *y*, *z*+1; (v) *x*, −*y*+1/2, *z*−1/2; (vi) −*x*+2, −*y*, −*z*+1; (vii) *x*+1, −*y*+1/2, *z*−1/2; (viii) −*x*+1, *y*+1/2, −*z*+3/2.
